# SwissRailNet: Four representations of a spatially embedded railway network dataset

**DOI:** 10.1016/j.dib.2025.112266

**Published:** 2025-11-12

**Authors:** Martin Sterchi, Emily Raubach, Lorenz Hilfiker

**Affiliations:** a School of Business, University of Applied Sciences and Arts Northwestern Switzerland FHNW, Riggenbachstrasse 16, 4600 Olten, Switzerland; b Institute of Psychology, University of Bern, Fabrikstrasse 8, 3012 Bern, Switzerland; c School of Education, University of Applied Sciences and Arts Northwestern Switzerland FHNW, Hofackerstrasse 30, 4132 Muttenz, Switzerland; d Institute of Geography, University of Bern, Hallerstrasse 12, 3012 Bern, Switzerland

**Keywords:** Static networks, Temporal networks, Spatial networks, Public transport

## Abstract

Public transport networks are frequently studied in the field of network science, including applications in epidemic modeling, infrastructure optimization, and machine learning. This paper introduces four representations of a Swiss railway network. The dataset is derived from open data sources of Swiss public transport companies and is the result of several data processing steps as well as a manual validation. In all four representations, nodes represent train stations, embedded in geographic space, making the network an example of a spatial network influenced by physical constraints such as mountains and lakes. The edge definitions vary, however. Edges connect (1) any pair of nodes with a train connection for which no train change is required, (2) any pair of nodes with a non-stop train connection, and (3) any pair of nodes that correspond to physically adjacent stations. The fourth representation corresponds to a temporal network in which each edge is attributed with the start and duration of a train connection.

Specifications Table

**Table:** 

Subject	Computer Sciences / Network Science
Specific subject area	Public transport networks
Type of data	Processed (.csv)
Data collection	The data have been collected from open data platforms of Swiss public transport companies. Subsequently, they have been processed, merged, and manually annotated and improved in order to fit common definitions of transport networks.
Data source location	The dataset introduced in this paper is derived from three publicly available datasets, accessible via https://opentransportdata.swiss/en/. The first dataset, called “Actual Data” (https://data.opentransportdata.swiss/en/dataset/istdaten), contains all scheduled stops for any mode of public transport on a given day. Each observation (row) corresponds to a scheduled stop of a public transport vehicle. For all four network representations introduced in this paper, we use data from Wednesday, March 5, 2025. The second dataset, “Service Points (Today)” (https://data.opentransportdata.swiss/en/dataset/service-points-actual-date), includes all valid service points as of March 5, 2025, most notably train stations with official names, geo-coordinates, and other information. The third dataset, “Number of Passengers Boarding and Alighting” (https://data.opentransportdata.swiss/en/dataset/einundaus), provides estimates on average passenger volumes at many (though not all) Swiss train stations. For computing the edge weights in one of the representations we use an additional dataset, called “Line (Operation Points)” (https://data.sbb.ch/explore/dataset/linie-mit-betriebspunkten/information/), containing geographical information on the physical railway lines and operation points along these lines.
Data accessibility	Repository name: Four representations of the Swiss railway network (data is shared under licence CC BY 4.0) Data identification number: 10.6084/m9.figshare.29609615.v2 Direct URL to data: https://doi.org/10.6084/m9.figshare.29609615.v2
Related research article	none

## 1 Value of the Data

• The dataset contains four different representations of the Swiss railway network, enabling a wide range of potential use cases and analyses. Researchers can select the representation that best fits their specific research question. Three of the four are static network representations, while the fourth is a temporal network representation, allowing both static and temporal network analyses. The static representations provide a real-world example of network layers that interpolate between planar infrastructure and small-world connectivity, all defined on the same set of nodes.
• The dataset provides a comprehensive representation of the Swiss railway system and follows a format commonly used in network science. Several publicly available datasets from open data platforms have been extensively processed and, for the first time, merged in order to offer a network dataset that is ready for a broad variety of analyses. Readily accessible network datasets are highly valued in the field, as researchers frequently seek to test hypotheses, validate models, or compare methods across diverse network topologies. The availability of well-documented networks enables more robust and reproducible research.
• The authors systematically reviewed the resulting network representations which led to the manual addition and deletion of a small number of edges, ensuring that the dataset accurately reflects reality and/or aligns with the network definitions used.
• The dataset can be reused for basic statistical analysis of network properties, which may yield interesting insights given the complexity and realism of the networks. More advanced applications include optimizing public transport, modeling dynamic processes on the network (e.g., random walks or epidemics), or using the data as input for graph machine learning tasks. For example, this dataset could be combined with observed train delay data to analyze how delays propagate through the network.
• The dataset is based on raw data extracted in March 2025. Given the stable nature of railway infrastructure and train schedules, we expect the dataset to remain valid for years to come. Nevertheless, we provide all code and references to the raw (source) data so that interested readers can fully replicate and/or update the dataset as needed.

## 2 Background

The motivation for preparing this dataset stems from the important role transport networks play in network science. For instance, several studies have investigated the spread of epidemics through global airline networks [[1], [2]]. Other studies have leveraged network data to analyze or optimize public transport infrastructure, many of them focusing on robustness [[3], [4], [5], [6], [7], [8], [9], [10], [11]]. Another focus has been the historical evolution of physical railway networks, particularly with respect to the French railway network [[12], [13], [14]].

Switzerland’s highly developed, complex railway network provides an interesting new use case for such types of analyses. Previous work on the Swiss public transport network [6,8] is outdated and does neither provide access to data nor documentation. We aim to fill this gap by presenting an updated, well-documented, and reproducible dataset.

We provide four distinct representations of the Swiss railway network based on different definitions of connectivity and enriched with travel and spatial attributes [15]. These representations support a range of different research questions and possible applications.

## 3 Data Description

The dataset [16] consists of seven comma-separated value (CSV) files, using semicolons (;) as separators. Following a standard structure in network science [17], we store nodes and their attributes in *nodelist.csv* and provide four CSV files for the edges in the four different representations. Moreover, we provide two additional CSV files containing the edges we manually removed from the *space-of-stations* representations and the edges we manually added to make all manual adjustments of that representation transparent. We refer the reader to the Experimental Design, Materials and Methods section for precise definitions of the four representations as well as a description of the data processing.

### 3.1 Nodes

*nodelist.csv* (189 KB) contains the following columns:

• BPUIC [integer]: Unique ID of a node (train station).
• STATION_NAME [string]: Official name of the train station.
• CANTON [string]: The (Swiss) canton the train station is located in.
• MUNICIPALITY [string]: The (Swiss) municipality the train station is located in.
• COMPANY [string]: The company that is responsible for the train station.
• LONGITUDE [decimal]: Longitude coordinate (WGS84).
• LATITUDE [decimal]: Latitude coordinate (WGS84).
• ELEVATION [decimal]: Elevation of the train station (in meters above sea level).
• AVG_DAILY_TRAFFIC [decimal]: Average daily traffic (Monday – Sunday) measured as the average number of passengers boarding and alighting trains at that station. Passengers in transit are counted twice. The numbers only reflect railway passengers and other types of public transport are not considered. For stations with low passenger volume, the number 49 is reported, which essentially indicates that the measurement is not reliable.
• AVG_DAILY_TRAFFIC_WEEKDAYS [decimal]: Average daily traffic (Monday – Friday), otherwise similar to the previous variable.
• AVG_DAILY_TRAFFIC_WEEKENDS [decimal]: Average daily traffic (Saturday – Sunday, public holidays), otherwise similar to the previous two variables.
• TRAFFIC_VALID [boolean]: false if average daily traffic information is unavailable or the value 49 is reported in any one of the three columns related to traffic; true otherwise.

The columns CANTON and MUNICIPALITY have 21 missing values across 19 train stations in Italy and 2 train stations in Germany. These stations appear in the data because they are operated by Swiss transport companies. Additionally, the three columns, AVG_DAILY_TRAFFIC, AVG_DAILY_TRAFFIC_WEEKDAYS, and AVG_DAILY_TRAFFIC_WEEKENDS, contain 496 missing values for train stations operated by smaller companies where no traffic measurements are available. The total number of nodes is 1626.

### 3.2 Edges

The following sections outline all files containing the edges of the network representations.

#### 3.2.1 Edges (Space-of-Changes)

*edgelist_SoCha.csv* (894 KB) contains the following columns:

• BPUIC1 [integer]: Node ID denoting the source of the (directed) edge.
• BPUIC2 [integer]: Node ID denoting the target of the (directed) edge.
• NUM_CONNECTIONS [integer]: Edge weight indicating the number of trains per day connecting the two nodes.
• AVG_DURATION [decimal]: Edge weight indicating the average time it takes to traverse that edge.

The total number of edges in this representation is 37,945. Notably, there are 26 edges for which AVG_DURATION is recorded as 0. These correspond to train connections that take less than one minute; since both departure and arrival times are rounded to the nearest minute, these connections are assigned a duration of 0 min.

#### 3.2.2 Edges (Space-of-stops)

*edgelist_SoSto.csv* (96 KB) contains the following columns:

• BPUIC1 [integer]: Node ID denoting the source of the (directed) edge.
• BPUIC2 [integer]: Node ID denoting the target of the (directed) edge.
• NUM_CONNECTIONS [integer]: Edge weight indicating the number of trains per day connecting the two nodes.
• AVG_DURATION [decimal]: Edge weight indicating the average time it takes to traverse that edge.

The total number of edges in this representation is 4211. Here, the same 26 edges as for the *space-of-changes* representation have a value of 0 for AVG_DURATION.

#### 3.2.3 Edges (Space-of-stations)

*edgelist_SoSta.csv* (44 KB) contains the following columns:

• BPUIC1 [integer]: Node ID denoting the source of the (undirected) edge.
• BPUIC2 [integer]: Node ID denoting the target of the (undirected) edge.
• DISTANCE_GEODESIC [decimal]: Geodesic distance between the two stations (in kilometers), computed based on the geo coordinates of the nodes.
• DISTANCE_EXACT [decimal]: Exact distance between the two stations along the railway tracks (in kilometers). This edge weight is only available for edges occurring in the source dataset called “Line (Operation Points)”.

The total number of edges in this representation is 1709. Two additional files, *edges_removed_SoSta.csv* and *edges_added_SoSta.csv*, contain the edges that were manually removed and added, respectively (in the same format as the primary edge list).

#### 3.2.4 Temporal edges

*edgelist_temporal.csv* (24.5 MB) contains the following columns:

• BPUIC1 [integer]: Node ID denoting the source of the (directed) edge.
• BPUIC2 [integer]: Node ID denoting the target of the (directed) edge.
• START [integer]: Start of the connection, measured in minutes after the start of the day (March 5, 2025, 00:00). The minimum value is 0 corresponding to trains departing exactly at midnight. The maximum value is 1955 corresponding to a night train departing from Basel SBB to Zürich HB at 08:35 on March 6, 2025. For reference, Switzerland observes Central European Time (CET; UTC+1) in winter and Central European Summer Time (CEST; UTC+2) in summer; since March 5, 2025 falls within the winter period, CET applies.
• DURATION [integer]: Duration of the connection, measured in minutes.

The total number of edges in this representation is 1110,766. As in the *space-of-changes* and *space-of-stops* representations, the same 26 node pairs consistently exhibit a DURATION value of 0 across all their temporal edges. Additionally, there are 4 further node pairs with DURATION values of 0, although these pairs display slightly higher AVG_DURATION values in the other two representations. In total, this results in 1084 edges with a DURATION of 0.

### 3.3 Data validation

The four network representations are all based on the same set of nodes and thus all consist of 1626 nodes. The three static representations consist of four disconnected components, each of which is strongly connected, meaning any pair of nodes within these components is connected by a (directed) path. The largest component contains 1592 nodes, while the smaller components range in size from 3 to 19 nodes. The smaller components have been validated manually and represent small, disconnected railway lines.

The most important network characteristics for the four representations are shown in Table 1. Note that for the computation of the diameter and the average shortest path length we focus on the giant connected component of the three static representations. The summary statistics are in good accordance with previous work on the Swiss railway network [6]. Comparing, for example, the average shortest path values to related work [6] (dating from 2006), we can observe an overall higher connectedness of the networks presented here. This can be explained by major infrastructure projects, such as the new Gotthard base tunnel, that have opened in the meantime. Overall, diameter and average shortest path values are rather large, which is typical for networks that reflect physical infrastructure embedded in the geographic space [6].

**Table 1:** Network characteristics of the four network representations. As is common in network science, the avg. degree is computed as # Edges / # Nodes for directed networks and (2 * # Edges) / # Nodes for undirected networks. The diameter and avg. shortest path length are computed based on the giant connected component.

	# Nodes	# Edges	Avg. degree	Diameter	Avg. shortest path length	Avg. clustering coefficient
*Space-of-changes*	1626	37,945	23.3	7	3.3	0.8798
*Space-of-stops*	1626	4211	2.6	46	11.9	0.1342
*Space-of-stations*	1626	1709	2.1	141	42.5	0.0019
*Temporal*	1626	1110,766	-	-	-	-

Given the regularity of train schedules, the *space-of-changes* and *space-of-stops* representations could also be considered undirected. In fact, for the *space-of-changes* representation only 969 edges are one-directional. Similarly, for the *space-of-stops* representation, only 93 are one-directional. Note, however, that bidirectional edges may have edge weights that differ slightly depending on directionality.

**Fig. 1:**
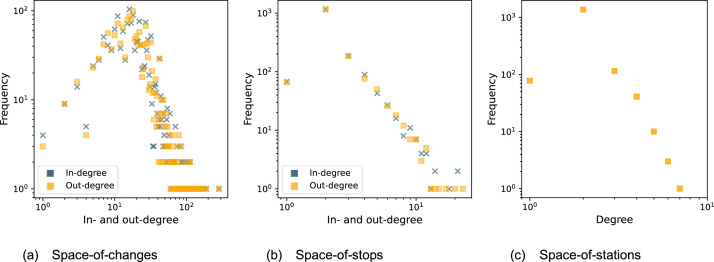
Degree distributions of the three static representations.

Fig. 1 shows the full degree distributions for the three static network representations on a log-log scale. The distributions look qualitatively similar to related work [6]. As expected, the station with the highest in- and out-degree values in all three representations is Zürich HB.

A comparison of the summary statistics for DURATION (in the *temporal* representation) and AVG_DURATION (in the *space-of-changes* representation) reveals substantial differences. For example, the mean DURATION is 25.67, whereas the mean AVG_DURATION is significantly higher at 33.82. This discrepancy is due to the method used to compute AVG_DURATION, which involves averaging the duration values for each node pair individually. Since the number of observations varies across node pairs, the overall mean of these per-pair averages results in unequal weighting of the underlying duration values. By contrast, the mean DURATION is calculated directly across all individual duration values, thereby assigning equal weight to each observation.

**Fig. 2:**
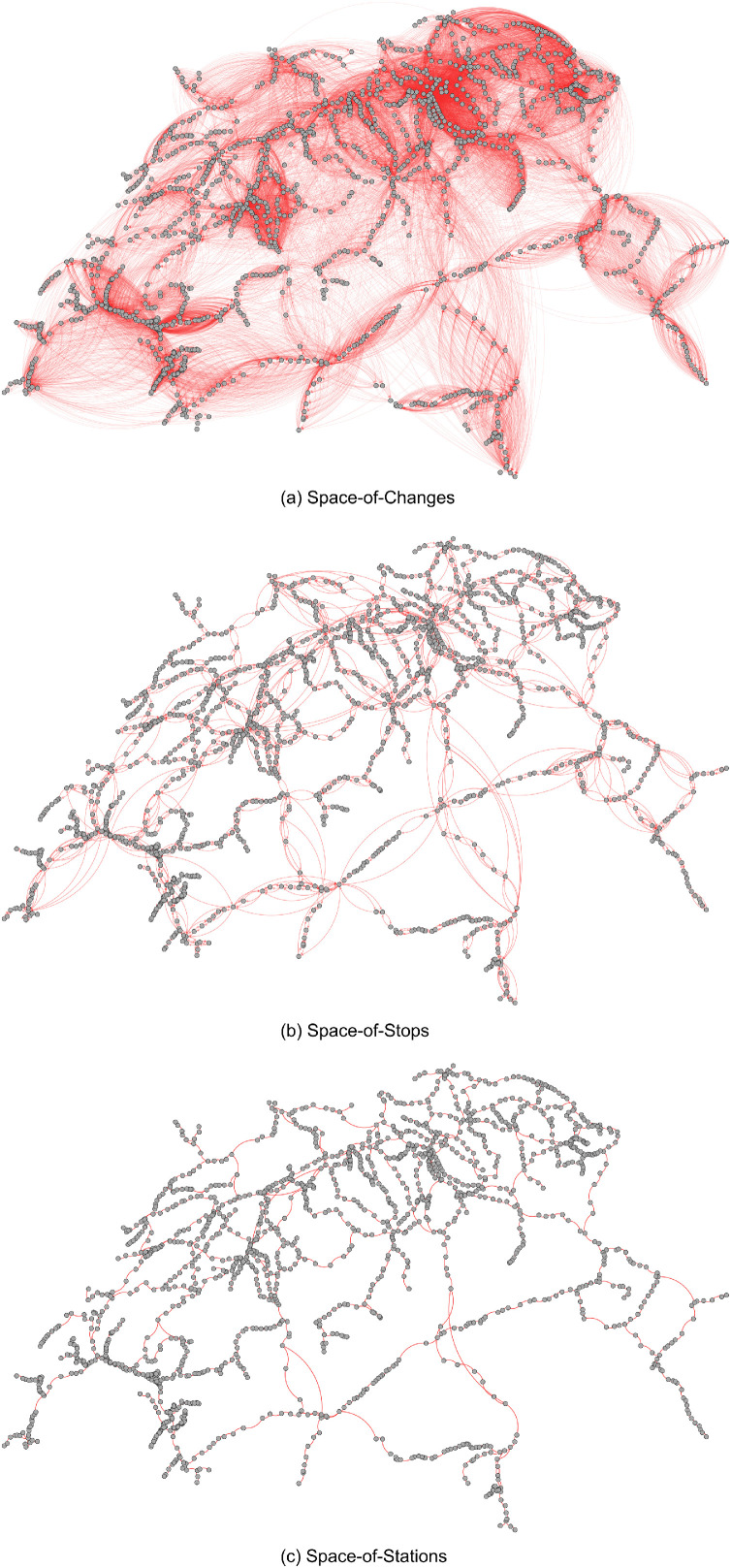
Visualization of the three static representations in which nodes are geographically positioned. For creating the visualizations, we used Gephi 0.10.

We performed extensive checks to assess the quality of the four representations. For example, we double checked for a sample of edges in the *space-of-changes* and the *space-of-stops* representations that the corresponding connections can be found in the official Swiss train schedule. Moreover, Fig. 2 shows the three static representations with nodes located according to their geo coordinates. For *space-of-stations* we visually checked that the edges in this representation match actual railway lines. Importantly, the plots hint at the geographic shape of Switzerland.

## 4 Experimental Design, Materials and Methods

In this section, we first elaborate on the four network representations that we use. In a second step, we outline the processing of the data. Our Python code as well as the source data can be found in a GitHub repository (https://github.com/martinSter/swiss-railway-networks).

### 4.1 Network representations

We present four different representations of the Swiss railway network, all based on the same set of nodes: namely, all active train stations in Switzerland. The key distinction between these representations lies in how the edges are defined. For three of the four representations, we follow definitions established in related work [6]. The fourth representation corresponds to a standard temporal network representation.

The first representation, known as *space-of-changes* [6], includes an edge between any two nodes that have a direct train connection, such that no change of trains is necessary. We adopt a directed version of this definition and further enrich the edges with two attributes: the number of connections per day and the average travel duration.

The second representation, *space-of-stops* [6], includes a directed edge between any two nodes that are connected by a direct, *nonstop* train. This representation is enhanced with the same two edge attributes as the *space-of-changes* representation.

The third representation, *space-of-stations* [6], includes an *undirected* edge for any two nodes that are directly connected by railway lines without any other station (node) in between and thus represents an approximation of the physical railway infrastructure. Each edge is augmented with the geodesic distance as a weight. In addition, for some edges an exact measurement of the distance along the railway tracks is also available. It is important to note that the notion of a physical railway network is not entirely well-defined at the level of granularity that we consider, where nodes represent stations, as this abstraction ignores essential infrastructure elements such as switches (e.g., locations where railway tracks diverge in different directions).

To illustrate these three static representations, consider a train line connecting Basel and Olten, with a stop in-between in Liestal. In the *space-of-changes* representation, this results in three directed edges: (Basel, Liestal), (Basel, Olten), and (Liestal, Olten). In the *space-of-stops* representation, only two directed edges exist: (Basel, Liestal) and (Liestal, Olten). In the *space-of-stations* representation, edges exist only between consecutive stations along the route, such as (Basel, Muttenz), (Muttenz, Liestal), etc.

The three static network representations differ in a notable way with respect to the interpretation of shortest path length [6]. In the *space-of-changes* representation, the shortest path length between two nodes reflects the minimum number of train changes required to travel from one to the other. In the *space-of-stops* representation, it reflects the number of train stops along the journey. Finally, in the *space-of-stations* representation, the shortest path length corresponds to the number of train stations passed along the route. From a graph-theoretic point of view, the three static network representations are related as follows: the *space-of-changes* representation contains the *space-of-stops* representation as a subgraph. In turn, the *space-of-stations* representation is approximately a (undirected) subgraph of the *space-of-stops* representation. The exact subgraph relationship is broken in this case because we do, in contrast with related work [6], manually complement the network with a number of edges to better reflect the actual physical railway network (e.g., to take physically existing shortcuts such as tunnels and high-speed lines into account).

Our fourth representation introduces temporal, directed edges, following the *space-of-changes* principle and is also based on the raw data for March 5, 2025. For any two nodes connected by a direct train, we record an edge along with the departure time of the connection and the duration required to traverse it. Importantly, this means that most node pairs are connected by multiple edges, each corresponding to a separate direct connection on that day. This temporal network representation is particularly useful for determining the fastest time-respecting paths between nodes which may help travelers identify the optimal route.

Importantly, our four network representations are also examples of *spatial* networks [15], where nodes and edges are embedded in geographic space. We account for this by providing geo-coordinates for all nodes in the network. The most important implication of this is that there are not only network topology constraints but also geographic constraints including distances and physical barriers such as mountains, lakes, and rivers.

### 4.2 Data processing

The dataset presented in this paper results from extensive processing of publicly available data provided by the Swiss transport authorities through the Open Data Platform Mobility Switzerland (ODPCH). The exact references to the source data are provided in the Specifications Table. The following subsections outline the key processing steps.

#### 4.2.1 Nodes

The procedure to create the node list is simple, as it only involves joining the information from the dataset called “Number of Passengers Boarding and Alighting” to the dataset called “Service Points (Today)”, which contains all currently active train stations. Note, however, that in three cases (the stations in Brig, Lugano, and Locarno) the data represents the physical station as two separate entities, due to different parts of the station being operated by different companies. From a network perspective, this is undesirable, so we merge the separate entities into a single node in each case.

Nodes are uniquely identified by a variable, called BPUIC. This ID variable is a combination of the country code (85 for Switzerland) and the station code from the International Union of Railways (UIC). BP is the abbreviation for the German word *Betriebspunkt*. For example, Basel SBB has the UIC station code 00010, making its BPUIC 8500010.

#### 4.2.2 Edges

The dataset “Actual Data”, containing all scheduled public transport trips on a given day, is the key dataset for extracting the edges for the four representations. In a first step, we filter that dataset down to include only train stops, reducing the number of observations from over 2.5 million to approximately 163,000. Some additional preprocessing involves removing a car shuttle train and a few train connections that were cancelled. The processing of the edges for the four representations is summarized in the following subsections.

##### 4.2.2.1 Space-of-changes

First, note that, in the dataset “Actual Data”, a sequence of train stops forms a trip, each uniquely identified by a trip identifier (FAHRT_BEZEICHNER). We group train stops by trip and sort them in ascending order based on departure time. Then, we iterate through the stops within each trip, extracting edges between a stop and all subsequent stops in that trip. For each extracted edge, we record the duration of the journey in minutes. Since multiple trains typically run between two stations on any given day, this process results in many duplicated edges (if durations are ignored). To address this, we aggregate all identical start-end point nodes, capturing the daily number of connections and the average travel time between each pair of stations.

##### 4.2.2.2 Space-of-stops

While the *space-of-changes* representation extracts edges for every stop and *all* subsequent stops in a trip, the *space-of-stops* representation instead only extracts an edge between each stop and its immediately following (consecutive) stop. As with the *space-of-changes* representation, we aggregate the extracted edges so that each (directed) pair of nodes appears only once. We manually remove the directed edges between Basel Bad Bahnhof and Schaffhausen, as there are several intermediate stops, all located in Germany, that are missing from the input dataset.

##### 4.2.2.3 Space-of-stations

This network representation is the most challenging to construct. We begin by converting the *space-of-stops* representation into an undirected graph. As in the related work [6], we then proceed to extract a subgraph by identifying and removing all edges that represent shortcuts, i.e., edges connecting two stations that are *not* physically consecutive. Specifically, we examine each edge to determine whether there is a trip that includes both nodes (stations) of the edge and, crucially, whether these two nodes are not consecutive within that trip. However, this method is susceptible to two types of errors.

First, the procedure may fail to detect a shortcut if the two stations do not appear together in any trip where the edge would function as a shortcut. A typical example of this problem is the shortcut edge between Bern and Zofingen: these two stations only occur together as part of a fast connection between Bern and Luzern. No other trip includes these two stations and, as a consequence, our procedure cannot identify it as a shortcut. Second, our procedure may miss certain edges if they are not captured in the source data (“Actual Data”) or it may incorrectly identify an edge as a shortcut, an error that mainly occurs in cases involving special infrastructure such as tunnels or high-speed lines. For instance, the stations Erstfeld and Biasca are connected via a path in the network with many stations in between but they should also be directly connected by an edge representing the Gotthard base tunnel, which opened in 2016. But our procedure is not able to capture that latter connection as no train stops consecutively at both stations.

To mitigate the first type of error, we conduct a manual review of all edges remaining after the procedure where the direct distance between the two stations is >10 km. This allows us to identify an additional 11 shortcuts which we manually remove from the network. To address the second type of error, we expand the network by explicitly adding 11 edges, some of them representing known special infrastructure projects. Note, however, that, as mentioned before, the manual addition of edges invalidates the initial subgraph relationship between the *space-of-stops* and the *space-of-stations* representations. Furthermore, we validate the resulting set of edges against the “Line (Operation Points)” dataset, which provides an overview of physical railway lines maintained by the Swiss Federal Railways (SBB), although it does not include infrastructure managed by other railway companies.

##### 4.2.2.4 Temporal edges

We extract the temporal edges according to the *space-of-changes* principle, meaning that the extraction procedure is essentially the same. For each edge, we record not only its duration but also its start time, expressed as the number of minutes elapsed since March 5, 2025, at 00:00 (midnight). Importantly, in this case, we do not aggregate edges that share the same start and end node pair, as retaining the full temporal detail of each individual connection is desired here.

## Limitations

We focus exclusively on Switzerland’s railway network and exclude other modes of public transport while other similar works [17] have considered several different modes of public transport. There are several reasons for this decision. First, the Swiss railway network is highly developed, with buses and trams mainly serving locally concentrated transportation needs (with the notable exception of *Postauto* buses in mountainous regions), or catering to tourism and leisure activities (e.g., boats and cable cars). Additionally, the only Swiss city with a small metro system is Lausanne. Second, Switzerland is a small country with a negligible number of domestic flights, primarily between Zürich and Geneva. Third, the data quality, homogeneity and availability, such as geo-coordinates of stations, decreases significantly when including other modes of transport. Consequently, we chose to focus solely on the Swiss public railway network.

Nevertheless, the network representations introduced here contain elements of multimodality. For instance, one might wish to distinguish edges based on the type of train connecting the nodes (e.g., intercity services vs. regional railway operations). Such information on train types is included in the raw “Actual Data,” and the processing routines can be adapted accordingly.

As mentioned above, the dataset presented here is based on raw data extracted in March 2025, and one might argue that it will soon be deprecated. However, the underlying infrastructure typically does not change rapidly. Moreover, in Switzerland, train schedules generally undergo only minimal year-to-year changes. Therefore, we are confident that the dataset will remain accurate for a long time. Nevertheless, we provide all our code and references to the source data in case anyone wishes to update the dataset with newer data.

## Ethics Statement

Our work did not involve human subjects or animals, or any data collected from social media platforms. We hereby confirm that we followed all ethical requirements set out by the journal.

## Declaration of Generative AI and AI-assisted Technologies in the Writing Process

During the preparation of this work the authors used ChatGPT in order to improve readability and flow of the article. After using this tool, the authors reviewed and edited the content as needed and take full responsibility for the content of the published article.

## CRediT Author Statement

**Martin Sterchi:** Conceptualization, Data curation, Software, Visualization, Writing – original draft. **Emily Raubach:** Conceptualization, Data Curation, Writing – review & editing. **Lorenz Hilfiker**: Conceptualization, Writing – review & editing.

## Data Availability

figshareFour representations of the Swiss railway network (Original data).
